# Polymorphisms of the *SCD1* Gene and Its Association Analysis with Carcass, Meat Quality, Adipogenic Traits, Fatty Acid Composition, and Milk Production Traits in Cattle

**DOI:** 10.3390/ani14121759

**Published:** 2024-06-11

**Authors:** Ruimin Liu, Xibi Fang, Xin Lu, Yue Liu, Yue Li, Xue Bai, Xiangbin Ding, Runjun Yang

**Affiliations:** 1 College of Animal Science, Jilin University, Changchun 130062, Chinaluxin20@mails.jlu.edu.cn (X.L.);; 2 Tianjin Key Laboratory of Agricultural Animal Breeding and Healthy Husbandry, College of Animal Science and Veterinary Medicine, Tianjin Agricultural University, Tianjin 300392, China

**Keywords:** Stearoyl-CoA desaturase-1 (*SCD1*), single nucleotide polymorphisms (SNPs), cattle, meat quality traits, milk traits

## Abstract

**Simple Summary:**

This study aimed to reveal the single nucleotide polymorphisms (SNPs) of the bovine Stearoyl-CoA desaturase-1 (*SCD1*) to explore the correlation between genotypes and carcass, meat quality, adipogenic traits, fatty acid composition, and milk production traits in cattle. Four SNPs, g.21272246 A>G, g.21272306 T>C, g.21272422 C>T, and g.21272529 A>G, were found by Sanger sequencing; further statistical analysis showed that these four SNPs of the *SCD1* gene were significantly associated with carcass traits and meat quality, including carcass weight, carcass fat coverage rate, rib eye area, marbling score, adipogenic traits, and fatty acid composition. Additionally, a modest effect on milk production traits, such as average milk yield and milk fat content, was observed in cows. Further haplotype analysis indicated that the combinations of H2H3 and H2H2 of SNPs had a higher value than others. Our results indicate that these four SNPs are potentially effective markers and could be used in marker-assisted breeding to improve meat and milk quality simultaneously in the future.

**Abstract:**

Stearoyl-CoA desaturase-1 (*SCD1*) is a key enzyme in the biosynthesis of monounsaturated fatty acids and is considered a candidate gene for improving milk and meat quality traits. Sanger sequencing was employed to investigate the genetic polymorphism of the fifth exon and intron of bovine *SCD1*, revealing four SNPs, g.21272246 A>G, g.21272306 T>C, g.21272422 C>T, and g.21272529 A>G. Further variance analysis and multiple comparisons were conducted to examine the relationship between variation sites and economic traits in Chinese Simmental cattle, as well as milk production traits in Holstein cows. The findings revealed these four loci exhibited significant associations with carcass traits (carcass weight, carcass length, backfat thickness, and waist meat thickness), meat quality (pH value, rib eye area, and marbling score), adipogenic traits (fat score and carcass fat coverage rate), and fatty acid composition (linoleic acid and α-linolenic acid). Furthermore, these loci were additionally found to be significantly associated with average milk yield and milk fat content in cows. In addition, a haplotype analysis of combinations of SNPs showed that H2H3 has a significant association with adipogenic traits and H2H2 was associated with higher levels of linoleic acid and α-linolenic acid than the other combinations. These results suggest that the four SNPs are expected to be prospective genetic markers for the above economic traits. In addition, the function of SNPs in exon 5 of *SCD1* on gene expression and protein structure needs to be explored in the future.

## 1. Introduction

Early in the 1950s, Simmental cattle were introduced to China and have become the dominant breed in the Chinese beef industry. Over time, through continuous breeding and adaptation improvements, Chinese Simmental cattle have achieved excellent growth, meat quality, and fat deposition performance, becoming an important breed with significant economic and agricultural value in China. Holstein cows were first introduced into China in the mid-19th century through hybridization with local cattle and long-term breeding, and they formed a better milk production performance, such as milk yield, milk fat content, and milk protein. Bovine milk is an important component of the human diet [1]. As a source of nutrients for human beings, milk is widely popular for its rich content of protein, calcium, zinc, and fatty acids. With the improvement of modern consumption levels and attention to the nutritional value of diet structure, the demand for the quality and quantity of meat and dairy products is also increasing.

Exploring molecular markers that can be used to assist in selecting economically important traits is crucial for advancing genetic improvement in cattle. Marker-assisted selection (MAS), one of the methods in molecular breeding, relies on nucleotide sequence variations among individuals that remain unaffected by environmental and other factors. This method enables direct selection at the DNA level during early stages, significantly reduces breeding time, and enhances efficiency. Single nucleotide polymorphisms (SNPs) are one of the most commonly used forms of genetic markers in genetic polymorphism studies. SNPs can be used as a molecular marker for assisted selection of the economic traits of cattle to analyze the correlation between the SNP locus and beef quality and milk production traits. Therefore, cattle with excellent genetic characteristics can be predicted and selected, providing an important scientific basis and technical means for the genetic improvement of beef cattle and dairy cows. SNP analysis has been widely applied in animal husbandry and agricultural research, providing important tools for molecular genetics in other fields [2].

Stearoyl-Coenzyme A desaturase (*SCD*) belongs to the family of fatty acid desaturases [3]. The *SCD1* gene is a key enzyme regulating fatty acid metabolism [4]. It catalyzes the conversion of saturated fatty acids (SFAs) to monounsaturated fatty acids (MUFAs), which affects the rate of fatty acid synthesis and fat deposition in animals. The fatty acid composition in muscle significantly impacts the flavor development of beef, with the content of unsaturated fatty acids directly influencing the taste of the meat [5]. As a critical enzyme in lipid metabolism, the bovine *SCD1* gene plays an important regulatory role in synthesizing unsaturated fatty acids. In cattle, the *SCD1* gene is primarily expressed in the mammary gland and adipose tissue. It is involved in cellular metabolism and the differentiation of precursor adipocytes, playing a role in regulating the composition of tissue fatty acids [6]. Furthermore, research on the association between the *SCD1* gene and meat quality suggests that *SCD1* may impact meat quality traits by influencing the shear force, marbling, and color [1,2,3]. As a result, the *SCD1* gene has emerged as an important candidate gene for MAS to improve beef quality. Moreover, studies on the *SCD1* gene in dairy cattle have revealed its crucial role in regulating the composition and content of milk fatty acids. It influences the quality, flavor, and nutritional value of milk and dairy products. The expression level of the *SCD1* gene is associated with the proportion of monounsaturated fatty acids in milk fat [1,7,8,9]. Studies on the *SCD1* gene encompass various aspects, including fatty acid metabolism, meat quality traits, and milk fatty acid composition. Further research is needed to explore the correlation between *SCD1* gene polymorphisms and important economic traits in cattle.

By detecting variations at single nucleotide sites and establishing SNP markers associated with specific traits, it becomes possible to better predict and select individuals with desirable traits, thereby accelerating the breeding process of livestock breeds. In this study, we used Sanger sequencing to screen potential genetic polymorphic loci in exons and introns of the *SCD1* gene. This study aimed to explore the correlation of different variation loci and genotypes with economic traits of cattle, including carcass traits, meat quality traits, fatty acid composition, and milk production traits and to clarify the potential roles of the *SCD1* gene in improving meat and milk quality traits.

## 2. Materials and Methods

### 2.1. Ethics Statements

Animal experiments were performed strictly following the guidance for the care and use of laboratory animals by the Jilin University Animal Care and Use Committee (Permit number: SYXK (Ji) 2008-0010/0011).

### 2.2. Animals and Sample Collection

A total of 334 Chinese Simmental steers (28 months old) were provided by the Inner Mongolian Baolongshan cattle farm (Tongliao, China) and were randomly selected from the offspring of a Simmental population of approximately 1000 female cows and 21 bulls. All individuals are similar in age, feeding conditions, and physical condition. Blood genomic DNA and tissue samples were extracted in our previous study [10] and stored at −80 °C in the laboratory for further use. After obtaining Dairy Herd Improvement (DHI) data, 73 cows were selected for jugular vein blood collection for subsequent experimental analysis.

### 2.3. Trait Analysis

Before slaughter, the live weight, the backfat thickness of living, and the area of *longissimus dorsi* muscle (by ultrasound) were measured and recorded; the weight of the carcass, omental fat, mesentery fat, and kidney fat were recorded at the slaughterhouse. After slaughter, the carcasses are stored in a refrigerated room at 0 to 4 °C for 24 h. All the measurements complied with the criterion GB/T 17238-2008 cutting standard of fresh and frozen beef of China (China Standard Publish) [11]. Furthermore, we also measured meat quality traits, including meat color and marbling, and recorded the fat coverage rate, marbling score, fat color score, muscle color score, rib eye area, and backfat thickness after slaughter. The day after slaughter, the 12th and 13th ribs’ *longissimus dorsi* muscle samples were collected from ripening carcasses and stored at −20 °C until they were thawed for fatty acid composition analysis and expressed as g/100 g fresh tissue [10,12].

In this study, 36 traits of carcass, meat quality, and adipogenic traits were obtained through measurement, and 14 fatty acid compositions were detected from the longissimus of the back, as described in our previous study [13]. Six milk composition traits, including milk yield, milk fat, milk protein percentage, lactose content, dry matter, and urea nitrogen, were detected and recorded for subsequent analysis.

### 2.4. Genomic DNA Extraction, PCR Amplification, and Identification

Genomic DNA was extracted from whole-blood samples (10 mL per cattle) using a blood DNA extraction kit (TIANGEN, Beijing, China), according to our previous reports. The purity and concentration of genomic DNA determined by NanoDrop 2000 (Thermo, Scientific, Waltham, MA, USA) showed an absorbance at OD_260 nm_/OD_280 nm_ between 1.8 and 2.0. In addition, the agarose gel electrophoresis detection showed genome DNA without RNA, protein, and ion pollution, which can be used for further experiments. Moreover, 45 randomly selected DNA samples of the detected population were mixed into a genomic DNA pool for a polymerase chain reaction (PCR) analysis to determine potential SNPs in the cattle *SCD1* gene. After obtaining the genetic variation loci in the detected populations, four SNPs located in exon 5 and intron 5 were selected for subsequent PCR, and 334 Chinese Simmental steers and 73 Holstein cattle were used as templates to amplify this polymorphic fragment, respectively.

The cattle *SCD1* gene sequence was obtained from the National Center for Biotechnology Information (NCBI) database query (GenBank: NC_037353.1), and 12 pairs of primers were designed by Primer Premier 6.0 software and synthesized by the Sangon Biotech company (Changchun, China), as shown in Table 1. The PCR reaction was performed in a 20 μL volume containing 1 μL genomic DNA, 0.4 μL primer (forward and reverse primer), 10 μL Green Taq Mix (2×), and 8.2 μL ddH_2_O. The PCR amplification procedure was performed as follows: pre-denaturation at 95 °C for 10 min, followed by 35 cycles of denaturation for 30 s at 95 °C, 55 °C for 30 s during annealing, extension for 30 s at 72 °C, and a final extension of 10 min at 72 °C, then cooling to 4 °C. Then, the PCR products were analyzed on a 1.5% agarose gel. The PCR products were sent to the Sangon Biotech company (Changchun, China) for Sanger sequencing by selecting clear single electrophoresis bands whose fragment sizes matched the expected product size.

The sequence data were compared with the reference sequences of the *SCD1* gene (NC_037353.1) by using SnapGene 4.1.9 (GSL Biotech LLC, Boston, MA, USA). Based on the sequencing results from PCR, amplifying the genomic DNA pool and individuals’ genomic sequences, the SNP loci and genotypes of the *SCD1* gene could be determined.

### 2.5. Statistical Analysis

Allele and genotype frequency were determined using relevant calculation formulas. The expected heterozygosity (He), effective number of alleles (Ne), and polymorphism information content (PIC) were calculated based on the genotyping results, which could estimate the degree of homozygosity or heterozygosity and the genetic polymorphism and test whether a genetic system is in equilibrium in a cattle population. The HaploView software 4.2 was also used to analyze the linkage disequilibrium of the SNP loci. A general linear model was adopted to analyze whether the genotypes of the different loci have synchronous effects on traits. We employed independent sample *t*-tests and an analysis of variance (ANOVA) in SPSS 25.0 (IBM, New York, NY, USA) to explore the correlations between the genotypes of different SNP loci in the *SCD1* gene and carcass traits, adipogenesis traits, meat quality traits, and fatty acid composition; finally, we used the LSD method for multiple comparisons, with a significance level of *p* < 0.05. We have presented the experimental data as the mean ± standard deviation. The model employed for this analysis was as follows:

Y*_ijk_* = u + ys*_i_* + m*_j_* + e*_ijk_*,

Y*_ijk_* represents the phenotypic observation of the *k*-th individual from the Simmental breed with genotype *j* in the *i*-th year season; u represents the population mean, ys*_i_* represents the year effect in the *i*-th year season, m*_j_* represents the effect of genotype *j*, and e*_ijk_* represents the random residual effect that correlates with the observed value [14].

## 3. Results

### 3.1. SNP Detection and Genotyping of the SCD1 Gene 

A total of 17 SNPs were detected in five PCR productions (*SCD1*-7, *SCD1*-8, *SCD1*-9, *SCD1*-10, *SCD1*-11) by DNA sequencing (Table 2). Previous research found that exon 5 and intron 5 amplified by the primer *SCD1*-8 have potential research significance. Therefore, as key loci for SNP screening, fragments from 334 Chinese Simmental steers and 73 Chinese Holstein cattle were amplified and genotyped.

**Figure 1:**
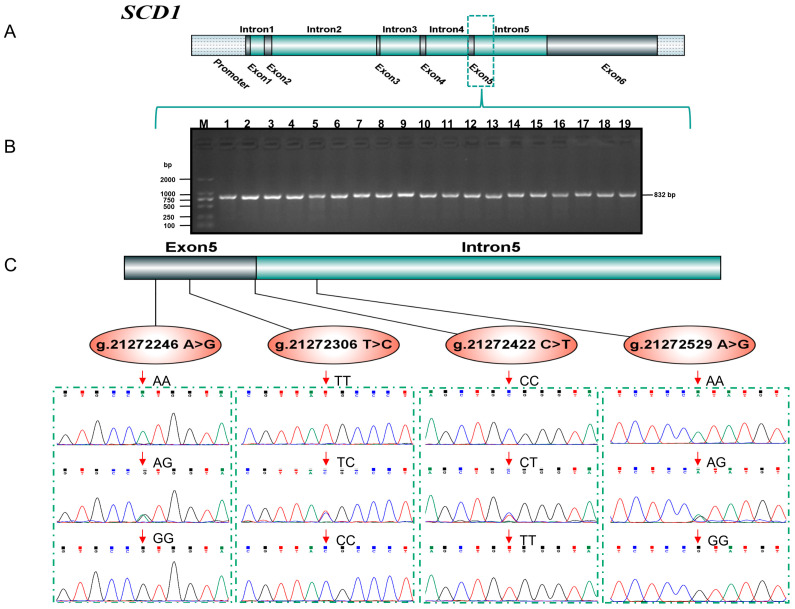
*SCD1* gene structure, agarose gel electrophoresis of PCR products, and SNPs detection. (**A**) Gene structure of *SCD1*; (**B**) Agarose gel electrophoresis of the *SCD1* gene PCR amplification product (partial) M: DL2000 Marker; 1–19: 832 bp PCR product of *SCD1*-8 primers; (**C**) Four SNPs’ detection and genotyping of the *SCD1* gene.

The electrophoresis results of the product showed that the bands that appeared on gel electrophoresis were consistent in size with the target fragment (832 bp) and had a good specificity (Figure 1B). Furthermore, based on the position of the bovine *SCD1* gene on the chromosome, these SNPs were marked in the genetic structure of *SCD1* (Figure 1A). The sequencing peak plot illustrated that four SNPs were screened in the fifth exon and intron of the *SCD1* gene, which were named separately as g.21272246 A>G, g.21272306 T>C, g.21272422 C>T, and g.21272529 A>G, and three genotypes were detected in this population (Figure 1C). Among these four SNPs, a missense mutation was detected at the locus g.21272422 C>T, while synonymous mutations were found at the other three loci. Specifically, a CGG to UGG missense mutation was recognized at the 293th codon in exon 5 (C>T) of *SCD1*, resulting in the replacement of alanine by valine (A293V), which is consistent with previous reports [4,5].

### 3.2. Analysis of Population Genetic Polymorphism, Linkage, and Haplotypes

**Figure 2:**
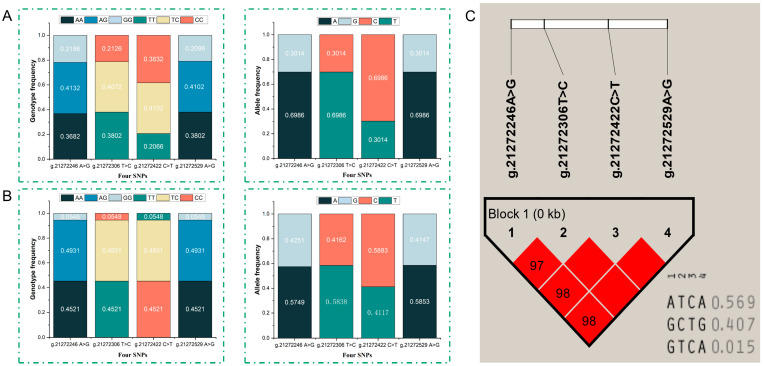
Genotypic and allelic frequencies of SNPs and the haplotypes composed of four SNPs of the *SCD1* gene. (**A**) Genotypic and allelic frequencies of SNPs in the *SCD1* gene in Chinese Simmental Steers; (**B**) Genotype frequency and allele frequency of SNPs in the *SCD1* gene in Chinese Holstein cattle; (**C**) The linkage disequilibrium and haplotypes frequencies in Chinese Simmental steers.

The genotypic and allelic frequencies and population genetic polymorphism of four SNPs were calculated and are shown in Table 3. At 21272246 A>G and g.21272529 A>G, the heterozygous genotype of AG was the dominant genotype, and allele A (0.575, 0.585) had a higher frequency than allele G (0.425, 0.415), respectively. At the g.21272306 T>C locus, allele T had a frequency of 0.584. Also, the heterozygous genotype TC was the dominant genotype and had a higher frequency (0.407) than the homozygote of TT (0.380) and CC (0.213). In addition, the CC, CT, and TT genotypes were observed for the g.21272422 C>T locus, allele C had a higher frequency (0.588) than allele T (0.412), and CT was the dominant genotype with a high frequency (0.410) (Figure 2A, Table 3). The genotypic frequencies of the four SNPs in the Chinese Holstein population were 0.452 for AA, 0.493 for AG, and 0.055 for GG, respectively. The genotypes of TT, TC/CT, and CC were also the same, the lowest genotype frequency of mutant homozygous was 0.055, and the heterozygous genotype was dominant accordingly. The allele frequency of A, T, and C was 0.699, higher than that of G, C, and T at 0.301 (Figure 2B, Table 3). The results showed that the homozygosity of the four SNPs was higher than the heterozygosity. In Chinese Simmental cattle, the polymorphic information contents of the four SNPs were 0.489, 0.486, 0.484, and 0.485, respectively, and this value was found to be 0.421 in Chinese Holstein cattle, which all belonged to a moderate polymorphic frequency (0.25 < PIC < 0.5). Additionally, there was a strong linkage between g.21272422 C>T and g.21272529 A>G (D′ = 1.0, LOD = 150.45, r^2^ = 0.988), g.21272306 T>C and g.21272529 A>G (D′ = 1.0, LOD = 153.03, r^2^ = 0.994), and g.21272306 T>C and g.21272422 C>T (D′ = 1.0, LOD = 148.70, r^2^ = 0.982).

The results of the haplotype analysis of the four SNPs of the *SCD1* gene were Hap1 (ATCA), Hap2 (GCTG), and Hap3 (GTCA). The haplotype frequencies of the three haplotypes were ATCA (0.569), GCTG (0.407), and GTCA (0.015), respectively. ATCA was the dominant haplotype (Figure 2C).

### 3.3. Association Analyses of SCD1 Gene Polymorphisms with Carcass and Meat Quality Traits in Chinese Simmental Cattle

Associations between the four SNPs and carcass traits, as well as meat quality traits results, are presented in Table 4. Significant associations were observed between the carcass weight, carcass length, backfat thickness, and waist meat thickness with the *SCD1* g.21272246 A>G, g.21272306 T>C, g.21272422 C>T, and g.21272529 A>G SNPs regarding the carcass. Further analysis of the genotypes revealed that individuals with the AG genotype had a higher carcass weight (*p* < 0.05) and backfat thickness (*p* < 0.01) than those individuals with the AA and GG genotype at g.21272246 A>G and g.21272529 A>G; however, the genotypic differences between AA and GG were not significant (*p* > 0.05). Similarly, individuals carrying the TC heterozygous genotype showed a higher carcass weight (*p* < 0.05) and backfat thickness (*p* < 0.01) than TT or CC homozygotes at g.21272306 T>C; at g.21272422 C>T, the individuals with the CT heterozygous genotype displayed an increased carcass weight (*p* < 0.05) and backfat thickness (*p* < 0.01) compared to CC or TT homozygous individuals, but no significant differences were detected between the CC and TT genotypes (*p* > 0.05). Waist meat thickness was significantly higher for the AG heterozygous individuals than those with the AA genotype (*p* < 0.05). In contrast, the carcass length of AA homozygous individuals was higher than that of GG homozygous individuals (*p* < 0.05) at g.21272246 A>G and g.21272529 A>G. In the same way, the waist meat thickness of individuals of the TC genotype was also higher than that of the TT genotype (*p* < 0.05), but the carcass length of TT homozygous individuals was higher than that of CC homozygous individuals (*p* < 0.05); at g.21272306 T>C, CT genotype individuals also had a higher value than that of CC genotype individuals (*p* < 0.05). In contrast, the carcass length of CC homozygous individuals was higher than that of TT homozygous individuals (*p* < 0.05) at g.21272422 C>T. Additionally, individuals with TC and CC genotypes showed a higher live weight than TT individuals at g.21272306 T>C (*p* < 0.05). A genotype comparison revealed no significant differences between individuals with the TC and CC genotypes (*p* > 0.05). AG genotype individuals had a higher live weight than AA and GG homozygous individuals at g.21272529 A>G (*p* < 0.05). Similarly, no significant associations were observed among individuals with the AA and GG genotypes (*p* > 0.05). No significant associations were observed between the g.21272246 A>G and g.21272422 C>T variants and the live weight measured (*p* > 0.05).

Similar, highly significant associations were also discovered in the meat quality traits at the four locations with the rib eye area, pH, and marbling score. Regarding the rib eye area, which is related to meat quality, it was observed that individuals with the AG genotype exhibited a higher rib eye area compared to those with GG genotype at g.21272246 A>G and g.21272529 A>G (*p* < 0.05). The rib eye area of TC heterozygous individuals was higher than that of CC individuals at g.21272306 T>C; the CT heterozygous individuals had a higher rib eye area than the individuals with the TT homozygous genotype (*p* < 0.05) at g.21272422 C>T, indicating a positive correlation between these genotypes and rib eye area related to meat quality traits. A highly significant correlation was observed with the meat quality trait of pH; individuals of the AA homozygous genotype had a significantly higher pH value at 0 h and 24 h compared with GG homozygous individuals at g.21272246 A>G and g.21272529 A>G (*p* < 0.01), and polymorphisms on g.21272306 T>C and g.21272422 C>T loci were also positively correlated with pH at 0 h and 24 h, respectively (*p* < 0.01). Surprisingly, the GG homozygous individuals displayed a higher marbling score than the AG heterozygous individuals g.21272246 A>G and g.21272529 A>G (*p* < 0.05), individuals with the CC genotype exhibited significantly higher levels compared to those with the TC heterozygous genotype at g.21272306 T>C, and TT homozygous individuals displayed a higher value than CT heterozygous individuals at g.21272422 C>T regarding marbling score, respectively (*p* < 0.01, *p* < 0.05).

### 3.4. Association Analyses of SCD1 Gene Polymorphisms with Adipogenesis Traits and Fatty Acid Composition

A correlation analysis of the SNPs in the *SCD1* gene related to adipogenesis traits and fatty acid composition is presented in Table 5. The individuals with the AA and AG genotypes had a significantly higher score of fat color than those of GG homozygous individuals at g.21272246 A>G and g.21272529 A>G. (*p* < 0.05). Moreover, at g.21272306 T>C, TT homozygous individuals’ scores were significantly higher than CC homozygous individuals (*p* < 0.05). Interestingly, the fat color score of CC genotype individuals had a highly significant difference compared to the TT genotype at g.21272422 C>T in the Chinese Simmental cattle population (*p* < 0.01). In carcass fat coverage rate, both heterozygous and mutant individuals at these four loci exhibited significantly higher levels than wild-type individuals (*p* < 0.01). Moreover, regarding the aspects of lung and trachea and kidney weight, the association analysis reflected that these metrics in heterozygous individuals were significantly higher than for homozygotes at the four SNPs of the *SCD1* gene (*p* < 0.01). A highly significant difference was observed in terms of cow penis weight at these four loci, wild-type individuals were higher in this metric than mutant individuals (*p* < 0.01). Heterozygous individuals had a higher spleen weight than that of homozygous individuals at g.21272422 C>T and g.21272529 A>G (*p* < 0.05). Furthermore, the renal adipose weight of heterozygous individuals was significantly higher than that of wild-type individuals at the g.21272306 T>C, g.21272422 C>T, and g.21272529 A>G (*p* < 0.01). In front hoof weight and heart weight, the individuals with the AG genotype had a higher weight of the front hoof and heart than those individuals with AA and GG genotypes at g.21272246 A>G; an identical pattern was observed at the remaining three loci.

As shown in Table 5, the SNPs of the *SCD1* gene had a significant association with linoleic acid and α-linolenic acid. In multiple genotype comparisons at g.21272246 A>G and g.21272529 A>G loci, GG genotype individuals exhibited a higher linoleic acid compared to AG and AA individuals (*p* < 0.01), and similar results were seen for the AA and AG genotypes (*p* > 0.05). Individuals of the CC genotype had a higher linoleic acid content than the TT and TC genotypes, and individuals of the TT genotype had a higher linoleic acid content than the CC and CT genotypes at g.21272306 T>C and g.21272422 C>T, respectively (*p* < 0.01). A higher α-linolenic acid content was significantly elevated in GG genotype individuals compared to that of the AA genotype at g.21272246 A>G and g.21272529 A>G (*p* < 0.01); at g.21272306 T>C locus, α-linolenic acid was significantly higher for CC genotype individuals than for the TT genotype, and TT genotype individuals had a higher α-linolenic acid content than the CC genotype at g.21272422 C>T (*p* < 0.01). It is worth mentioning that a significant association was observed with stearic acid, wherein CC individuals displayed a higher stearic acid content than TT and TC genotype individuals at g.21272306 T>C (*p* < 0.05). No significant associations were observed between the three other loci and stearic acid (*p* > 0.05).

### 3.5. Association Analysis of the SNPs in the SCD1 Gene with Milk Production Traits in Chinese Holstein Cows

The association analysis is shown in Table 6. These four SNPs had a significant association with the average milk yield and milk fat content. The average milk yield of the GG genotype individuals was significantly higher compared to that of AG individuals at g.21272246 A>G and g.21272529 A>G (*p* < 0.05). Individuals of the CC genotype had a higher average milk yield than the TC genotype at g.21272306 T>C, and the CC genotype individuals had a higher average milk yield than the TC genotype at g.21272422 C>T, respectively (*p* < 0.05). Additionally, individuals with the AA genotype had a higher milk fat content than those with the GG genotype at g.21272246 A>G and g.21272529 A>G (*p* < 0.05). The milk fat content of the TT genotype individuals was higher than that of CC homozygous individuals (*p* < 0.05) at g.21272306 T>C, and CC genotype individuals also had a higher value than that of the TT genotype at g.21272422 C>T in Chinese Holstein cows (*p* < 0.05).

### 3.6. Association Analysis of Haplotypes and Carcass Traits, Meat Quality, Adipogenesis Traits, and Fatty Acid Composition in Chinese Simmental Cattle

The haplotype analysis results have been described above (Figure 2C). Five haplotype combinations, H1H1, H1H2, H1H3, H2H2, and H2H3, were reconstructed in Chinese Simmental steers as illustrated in Table 7. The correlation analysis indicates that the haplotype combination H2H3 demonstrates significantly higher values for carcass fat coverage rate, renal adipose weight, oxtail weight, and muscle color score compared to the H1H1, H1H2, H1H3, and H2H2 haplotypes (*p* < 0.05), but there is no significant association in the other haplotype combinations. Since the haplotype combinations of H2H3 represented too few individuals to be significant in the statistical analysis, the subsequent correlation analysis between these four haplotype combinations and fatty acid composition is presented in Table 8. The H2H2 haplotype combination exhibits significantly higher levels of linoleic acid and α-linolenic acid than H1H1 and H1H2, while it is similar to the H1H1 and H1H2 haplotype combination individuals (*p* > 0.05).

## 4. Discussion

In recent years, with the increasing emphasis on healthy eating and the demand for nutrient-rich ingredients, beef and milk as high-quality protein sources have received much attention. Despite the general focus on production and reproductive traits, carcass and meat quality characteristics after slaughter, as well as milk production traits of cows, are more critical factors in determining the success of beef and dairy cattle. As a standard molecular marker, SNPs play an essential role in genetics and molecular breeding and can be used to explore the association between genes and economic traits. SNP association analysis can reveal the genetic influence on economic traits, which can guide the optimization of breed improvement and enhance the effect of economic traits in the selection process [15]. Therefore, it is necessary to select appropriate means to promote the economic traits of improving meat and milk quality.

The *SCD1* gene is abundantly expressed in adipose tissue, liver, and skeletal muscle [16], playing an essential role in the biosynthesis of unsaturated fatty acids in the milk and meat of cattle, and its polymorphisms are frequently considered as an influencing factor [17]. Based on previous reports, the present study identified four known SNPs, g.21272246 A>G, g.21272306 T>C, g.21272422 C>T, and g.21272529 A>G, in the fifth exon and intron of the bovine *SCD1* gene. Three genotypes were found to be present by DNA sequencing, and a missense mutation was found at g.21272422 C>T in exon 5, resulting in a CGG to UGG change at codon 293. This locus has been reported to be significantly associated with marbling and rib thickness in Japanese Black cattle [18], consistent with the association between SNP and marbling in the present study. However, no association was observed for carcass traits measured in northern Australian crossbreed beef cattle [19]. Additionally, the content of monounsaturated fatty acids in milk or meat products was associated with the SCD gene SNP rs41255693 (g.21272422 C>T) in cattle [20,21].

Further research is required to investigate the relationship between SNPs and the carcass traits, meat quality traits, adipogenic traits, and fatty acid composition of Chinese Simmental cattle, as well as their association with the milk production traits of Chinese Holstein cows. The association analyses revealed that four SNPs were significantly associated with carcass traits (carcass weight, carcass length, and backfat thickness, etc.), meat quality traits (marbling score, pH, rib eye area, etc.), adipogenic traits (fat color, fat coverage rate, etc.), and fatty acid composition (linoleic acid, α-linolenic acid) in Chinese Simmental steers. Additionally, these SNPs were correlated with milk production traits for Chinese Holstein cows, for instance, average milk yield and milk fat content. These findings provide valuable insights into the genetic basis of economically important traits and may have implications for breeding programs to improve livestock productivity.

Haplotypes were also examined to establish relationships with these significant traits. The analysis showed that the H2H3 haplotype had higher values for certain traits (fat coverage rate, muscle color score, etc.) in Chinese Simmental cattle, while the H2H2 haplotype exhibited superior linoleic acid and α-linolenic acid compared to H1H1 and H1H2 in Chinese Holstein cows. Consequently, these four SNPs could be utilized to select individuals with those combinations of haplotypes.

The *SCD1* gene regulates the synthesis of unsaturated fatty acids. Previous studies have shown that fatty acids play an important role in the flavor of ruminant meat [22] and are also key in the fatty acid composition of adipose tissue and animal products such as meat and milk [14]. Smith et al. showed that the flavor produced by beef is related to the composition and type of fatty acids, and the high content of unsaturated fatty acids in fat is more conducive to producing a good flavor [23]. Kim et al. found a significant association between the SNPs of *SCD* and saturated and unsaturated fatty acids in Hanwoo steers [24]. Taniguchi et al. suggested that the *SCD* gene was responsible for the genetic variation in fatty acid composition in steers of the Japanese Black breed [25]. Linoleic acid is an essential fatty acid that can only be obtained from the diet [26]. In this study, we were surprised that four SNPs were statistically significant with linoleic acid and α-linolenic acid content in Chinese Simmental cattle. Our findings imply that these loci in the *SCD1* gene may serve as potential molecular markers to improve fatty acid composition selection in beef cattle.

In addition to its role in carcass, meat quality, and fatty acid composition, the *SCD1* gene also plays a crucial role in determining milk production traits. Studies have shown that the *SCD1* gene affects the fatty acid profile of milk [27,28]. To further explore the impact of SNPs on milk traits, our study was conducted on a population of dairy cattle, and preliminary correlation analysis showed significant associations between the four SNPs and the average milk yield and milk fat content of dairy cows.

Macciotta et al. found that SNPs in exon 5 of the *SCD1* gene were associated with milk production and protein production in Italian Holstein cattle [29]. The influence of the A293V SNP in the *SCD1* gene on milk traits in cattle has been extensively studied [30,31]. Regarding A293V SNP, at locus g.21272422 C>T, resulting in a substitution of alanine by valine at position 293 of the *SCD1* protein, individuals of the TT genotype had a significantly higher average milk yield compared to the CC genotype; our findings support the assertion that the A293V SNP has a positive impact on milk production traits in cattle, and these mutations in exons and introns may be potential genetic markers for improving milk production ability in Chinese Holstein cows. Of course, future studies should confirm these associations in larger cattle populations. Additionally, the effects of SNPs in exon 5 of *SCD1* on gene expression and protein structures need to be further explored. 

## 5. Conclusions

This study identified four SNPs in the fifth exon and intron of the *SCD1* gene. Subsequently, their association with economic traits was further analyzed in Chinese Simmental steers and Holstein cows. The study revealed that the four SNPs of the *SCD1* gene were associated with carcass traits, meat quality, adipogenic traits, and fatty acid composition in beef cattle. Additionally, we observed a modest effect on milk production traits such as average milk yield and milk fat content in cows, especially at locus g.21272422 C>T. Further haplotype analysis indicated that H2H3 and H2H2 had better value than the others. Furthermore, the significance of the well-known *SCD1* gene marker in bovine breed improvement is underscored. These findings could serve as a valuable reference for further exploration into the subsequent functionality and mechanism of the *SCD1* gene.

## Figures and Tables

**Table 1:** PCR primer sequences of the *SCD1* gene.

Primer Names	Primer Sequences (5′-3′)	Product Size (bp)	Tm (°C)
*SCD1*-1	F: TAGTGGGTGACACATTCATAGC R: GGATTGCCTGGGAGGATGA	833	55
*SCD1*-2	F: AATCATCCTCCCAGGCAATCC R: GCGTAAGAGGTTCAGCCAATG	702	55
*SCD1*-3	F: CGGGTTTGAGGACACGTCT R: TTTATTCGTTGCCAACAAGGG	599	55
*SCD1*-4	F: TGTGCAGCATCCAGTTCTTG R: AAGGCGGAAGACAGGGAAG	781	54
*SCD1*-5	F: ATCTCTAGCTCCTACACAACCA R: AGCCCTCTAAAGTCACTCATCT	704	54
*SCD1*-6	F: AGGTTAGCAGAAGGTCAGAGG R: AAGACCACAACAGCCAGACT	746	55
*SCD1*-7	F: GCATTCCACTCACCACATAACC R: TTGTGCCTCTCCTCGCTATG	936	55
*SCD1*-8	F: TCCTTGCTCCACCACTTCC R: CCACCCAGATGACCCTACTC	832	55
*SCD1*-9	F: CATTCATTCAACAGCAACAGGT R: CAGGAGAGAAAGGGAGCATACT	1002	54
*SCD1*-10	F: CTCCCTTTCTCTCCTGACTCTG R: CCATCACTGCCTCTGAATACAC	1002	55
*SCD1*-11	F: TCACTGAACCACTGTTTCTCTT R: AAGGCATCCAGATAAGTTGTCA	1203	51
*SCD1*-12	F: ATGCTGACAACTTATCTGGATG R: CAGGGCAATCAGATTCACTTT	981	51

**Table 2:** 17 SNPs’ information regarding the *SCD1* gene.

Primer Names	SNP	Mutation Region	Number	Variation ID	Gene Position
***SCD1*** **-7**	A>G	intron3	2	rs41255689	26:21270336
	A>G	intron4		rs41255690	26:21270739
***SCD1*** **-8**	A>G	exon5	4	rs41255691	26:21272246
	T>C	exon5		rs41255692	26:21272306
	C>T	exon5		rs41255693	26:21272422
	A>G	intron5		rs383175036	26:21272529
***SCD1*** **-9**	G>T	exon6	3	rs41255694	26:21275659
	G>C	exon6		rs41255695	26:21275732
	C>A	exon6		rs41255696	26:21275851
***SCD1*** **-10**	C>T	exon6	2	rs41255697	26:21276141
	A>G	exon6		rs41255698	26:21276672
***SCD1*** **-11**	A>G	exon6	6	rs41255700	26:21277095
	C>T	exon6		rs41255701	26:21277195
	G>A	exon6		rs41255702	26:21277296
	T>G	exon6		rs41255703	26:21277378
	G>A	exon6		rs41255704	26:21277585
	G>A	exon6		rs382676818	26:21277770

**Table 3:** Genetic diversities of the *SCD1* gene in Chinese Simmental steer and Chinese Holstein cattle.

SNP	g.21272246 A>G				g.21272306 T>C				g.21272422 C>T				g.21272529 A>G			
Gene position	26:21272246				26:21272306				26:21272422				26:21272529			
Mutation type	synonymous mutation				synonymous mutation				missense mutation				synonymous mutation			
Breed	Chinese Simmental steer *n =* 334		Chinese Holstein cattle *n =* 73		Chinese Simmental steer *n =* 334		Chinese Holstein cattle *n =* 73		Chinese Simmental steer *n =* 334		Chinese Holstein cattle *n =* 73		Chinese Simmental steer *n =* 334		Chinese Holstein cattle *n =* 73	
Genotype frequency	AA (123)	0.368	AA (33)	0.452	TT (127)	0.380	TT (33)	0.452	CC (128)	0.383	TT (33)	0.452	AA (127)	0.380	AA (33)	0.452
	AG (138)	0.413	AG (36)	0.493	TC (136)	0.407	TC (36)	0.493	CT (137)	0.410	TC (36)	0.493	AG (137)	0.410	AG (36)	0.493
	GG (73)	0.219	GG (4)	0.055	CC (71)	0.213	CC (4)	0.055	TT (69)	0.207	CC (4)	0.055	GG (70)	0.210	GG (4)	0.055
Allele frequency	A	0.575	A	0.699	T	0.584	T	0.699	C	0.588	C	0.699	A	0.585	A	0.699
	G	0.425	G	0.301	C	0.416	C	0.301	T	0.412	T	0.301	G	0.415	G	0.301
He	0.413		0.493		0.407		0.493		0.410		0.493		0.410		0.493	
PIC	0.489		0.421		0.486		0.421		0.484		0.421		0.485		0.421	
Ne	1.704		1.972		1.686		1.972		1.695		1.972		1.695		1.972	

Note: He, gene heterozygosity; PIC, polymorphic information content; Ne, effective number of alleles.

**Table 4:** Association analyses of *SCD1* gene polymorphisms with economic traits in Chinese Simmental cattle.

Traits		g.21272246 A>G			g.21272306 T>C			g.21272422 C>T			g.21272529 A>G		
		AA (*n* = 123)	AG (*n =* 123)	GG (*n =* 73)	TT (*n =* 123)	TC (*n =* 123)	CC (*n =* 71)	CC (*n =* 128)	CT (*n =* 123)	TT (*n =* 67)	AA (*n =* 127)	AG (*n =* 137)	GG (*n =* 70)
		Mean ± SD	Mean ± SD	Mean ± SD	Mean ± SD	Mean ± SD	Mean ± SD	Mean ± SD	Mean ± SD	Mean ± SD	Mean ± SD	Mean ± SD	Mean ± SD
Carcass traits	LW^1^ (kg)	487.73 ± 58.66	500.31 ± 58.54	487.52 ± 64.2	486.73 ± 57.83 ^b^	501.28 ± 57.29 ^a^	488.69 ± 67.38 ^a^	486.36 ± 57.65	501.78 ± 59.05	487.32 ± 64.67	486.24 ± 57.86 ^b^	502.41 ± 58.59 ^a^	486.30 ± 64.76 ^b^
	CW (kg)	254.11 ± 35.34 ^b^	262.81 ± 36.77 ^a^	253.52 ± 41.81 ^b^	253.7 ± 35.03 ^b^	263.51 ± 36.22 ^a^	253.68 ± 43.05 ^b^	253.50 ± 34.95 ^b^	263.68 ± 37.05 ^a^	253.01 ± 41.89 ^b^	253.39 ± 35.07 ^b^	264.11 ± 36.75 ^a^	252.38 ± 41.92 ^b^
	DP (%)	52.02 ± 2.30	52.42 ± 2.15	51.82 ± 2.86	52.05 ± 2.31	52.46 ± 2.15	51.73 ± 2.85	52.04 ± 2.30	52.44 ± 2.15	51.74 ± 2.88	52.03 ± 2.30	52.47 ± 2.14	51.72 ± 2.86
	CL (cm)	140.52 ± 8.13 ^a^	139.42 ± 8.36	138.19 ± 7.90 ^b^	140.34 ± 8.15 ^a^	139.50 ± 8.23	138.23 ± 8.22 ^b^	140.44 ± 8.14 ^a^	139.57 ± 8.33	137.90 ± 7.90 ^b^	140.36 ± 8.12 ^a^	139.65 ± 8.38	137.91 ± 7.84 ^b^
	CD (cm)	64.78 ± 3.14	64.32 ± 3.48	64.14 ± 3.33	64.67 ± 3.09	64.38 ± 3.43	64.17 ± 3.56	64.73 ± 3.11	64.42 ± 3.49	63.99 ± 3.35	64.69 ± 3.07	64.45 ± 3.53	64.01 ± 3.34
	CBD (cm)	65.33 ± 3.95	65.45 ± 3.47	64.91 ± 3.62	65.21 ± 3.89	65.48 ± 3.38	65.03 ± 3.88	65.27 ± 3.90	65.53 ± 3.46	64.84 ± 3.69	65.23 ± 3.88	65.56 ± 3.49	64.87 ± 3.67
	HLC (cm)	48.72 ± 4.08	48.97 ± 3.45	49.06 ± 3.56	48.73 ± 4.03	48.99 ± 3.42	49.07 ± 3.68	48.72 ± 4.02	49.02 ± 3.45	48.97 ± 3.66	48.70 ± 4.03	49.03 ± 3.45	48.99 ± 3.63
	HLW (cm)	44.56 ± 2.92	44.85 ± 2.56	44.56 ± 2.71	44.49 ± 2.92	44.86 ± 2.51	44.73 ± 2.74	44.49 ± 2.94	44.87 ± 2.51	44.66 ± 2.72	44.46 ± 2.92	44.89 ± 2.53	44.66 ± 2.7
	HLL (cm)	80.22 ± 4.40	80.17 ± 4.01	80.01 ± 3.95	80.12 ± 4.47	80.12 ± 3.94	80.24 ± 3.95	80.19 ± 4.48	80.14 ± 3.92	80.11 ± 3.92	80.14 ± 4.46	80.16 ± 3.95	80.16 ± 3.92
	TMT (cm)	17.81 ± 1.81	17.88 ± 1.68	18.20 ± 1.58	17.79 ± 1.82	17.88 ± 1.69	18.29 ± 1.50	17.76 ± 1.81	17.91 ± 1.70	18.26 ± 1.48	17.77 ± 1.82	17.90 ± 1.70	18.25 ± 1.48
	BFT (cm)	0.93 ± 0.61 ^B^	1.10 ± 0.64 ^A^	0.93 ± 0.60 ^B^	0.96 ± 0.63 ^B^	1.10 ± 0.63 ^A^	0.87 ± 0.57 ^B^	0.94 ± 0.63 ^B^	1.10 ± 0.63 ^A^	0.89 ± 0.57 ^B^	0.95 ± 0.63 ^B^	1.10 ± 0.63 ^A^	0.88 ± 0.57 ^B^
	WMT (cm)	6.77 ± 0.92 ^b^	6.94 ± 0.85 ^a^	6.91 ± 0.93 ^ab^	6.78 ± 0.91 ^b^	6.97 ± 0.87 ^a^	6.89 ± 0.90 ^ab^	6.76 ± 0.91 ^b^	6.96 ± 0.87 ^a^	6.91 ± 0.90 ^ab^	6.77 ± 0.91 ^b^	6.97 ± 0.87 ^a^	6.89 ± 0.90 ^ab^
Meat quality traits	pH (0 h)	6.33 ± 0.51 ^A^	6.19 ± 0.49 ^B^	6.16 ± 0.51 ^B^	6.31 ± 0.51 ^A^	6.18 ± 0.49 ^B^	6.18 ± 0.52 ^B^	6.32 ± 0.51 ^A^	6.19 ± 0.49 ^B^	6.17 ± 0.52 ^B^	6.32 ± 0.51 ^A^	6.19 ± 0.49 ^B^	6.17 ± 0.51 ^B^
	pH (24 h)	5.60 ± 0.34 ^A^	5.55 ± 0.31 ^AB^	5.49 ± 0.37 ^B^	5.60 ± 0.34 ^A^	5.55 ± 0.31 ^AB^	5.49 ± 0.38 ^B^	5.60 ± 0.33 ^A^	5.55 ± 0.31 ^AB^	5.48 ± 0.38 ^B^	5.60 ± 0.33 ^A^	5.55 ± 0.31 ^AB^	5.49 ± 0.38 ^B^
	MBS	5.39 ± 0.72 ^ab^	5.26 ± 0.74 ^b^	5.48 ± 0.69 ^a^	5.40 ± 0.72 ^AB^	5.24 ± 0.75 ^B^	5.49 ± 0.67 ^A^	5.40 ± 0.71 ^ab^	5.26 ± 0.75 ^b^	5.48 ± 0.68 ^a^	5.40 ± 0.72 ^ab^	5.25 ± 0.75 ^b^	5.49 ± 0.68 ^a^
	MCS	5.72 ± 1.08	5.57 ± 1.08	5.64 ± 1.12	5.70 ± 1.08	5.62 ± 1.10	5.58 ± 1.10	5.70 ± 1.07	5.61 ± 1.09	5.59 ± 1.12	5.70 ± 1.07	5.61 ± 1.09	5.59 ± 1.11
	REA (cm^2^)	79.28 ± 13.44 ^ab^	81.30 ± 12.56 ^a^	77.23 ± 11.88 ^b^	79.21 ± 13.28 ^ab^	81.12 ± 12.69 ^a^	77.73 ± 12.11 ^b^	79.18 ± 13.17 ^ab^	81.07 ± 12.8 ^a^	77.78 ± 11.98 ^b^	79.19 ± 13.23 ^ab^	81.20 ± 12.68 ^a^	77.53 ± 12.08 ^b^

The different letters (a,b) indicate significant differences among the genotypes (*p* < 0.05); The different letters (A,B) indicate highly significant differences among the genotypes (*p* < 0.01); Mean ± SD, mean ± standard deviation. LW^1^, live weight; CW, carcass weight; DP, dressing percentage; CL, carcass length; CD, carcass depth; CBD, carcass breast depth; HLC, hind legs’ circumference; HLW, hind legs’ width; HLL, hind legs’ length; TMT, thigh meat thickness; BFT, backfat thickness; WMT, waist meat thickness; pH (0 h), beef pH value after slaughter; pH (24 h), beef pH value 24 h after degassing; MBS, marbling score (the score range of marbling is from No. 1 to 9); MCS, muscle color score (the score range of muscle color is from No. 1 to No. 7); REA, rib eye area.

**Table 5:** Association analyses of *SCD1* gene polymorphisms with adipogenesis traits and fatty acid composition in Chinese Simmental cattle.

Traits		g.21272246 A>G			g.21272306 T>C			g.21272422 C>T			g.21272529 A>G		
		AA (*n =* 123)	AG (*n*= 138)	GG (*n =* 73)	TT (*n =* 127)	TC (*n =* 136)	CC (*n =* 71)	CC (*n =* 128)	CT (*n =* 137)	TT (*n =* 69)	AA (*n =* 127)	AG (*n =* 137)	GG (*n =* 70)
		Mean ± SD	Mean ± SD	Mean ± SD	Mean ± SD	Mean ± SD	Mean ± SD	Mean ± SD	Mean ± SD	Mean ± SD	Mean ± SD	Mean ± SD	Mean ± SD
Adipogenesis traits	MBS	5.39 ± 0.72 ^ab^	5.26 ± 0.74 ^b^	5.48 ± 0.69 ^a^	5.40 ± 0.72 ^AB^	5.24 ± 0.75 ^B^	5.49 ± 0.67 ^A^	5.40 ± 0.71 ^ab^	5.26 ± 0.75 ^b^	5.48 ± 0.68 ^a^	5.40 ± 0.72 ^AB^	5.25 ± 0.75 ^B^	5.49 ± 0.68 ^A^
	FCS	2.79 ± 1.03 ^a^	2.77 ± 0.87 ^a^	2.53 ± 1.04 ^b^	2.79 ± 1.02 ^a^	2.75 ± 0.85	2.55 ± 1.08 ^b^	2.80 ± 1.02 ^A^	2.77 ± 0.87 ^a^	2.49 ± 1.04 ^B,b^	2.80 ± 1.02 ^a^	2.76 ± 0.85 ^a^	2.53 ± 1.07 ^b^
	BFT (cm)	0.93 ± 0.61 ^B^	1.10 ± 0.64 ^A^	0.93 ± 0.60 ^B^	0.96 ± 0.63 ^B^	1.10 ± 0.63 ^A^	0.87 ± 0.57 ^B^	0.94 ± 0.63 ^B^	1.10 ± 0.63 ^A^	0.89 ± 0.57 ^B^	0.95 ± 0.63 ^B^	1.10 ± 0.63 ^A^	0.88 ± 0.57 ^B^
	FCR%	47.88 ± 21.66 ^b^	50.44 ± 20.91 ^a^	51.16 ± 19.93 ^a^	48.16 ± 21.5 ^B^	51.21 ± 20.99 ^A^	49.8 ± 19.78 ^AB^	47.7 ± 21.65 ^B^	51.09 ± 20.95 ^A^	50.43 ± 19.67 ^AB^	47.91 ± 21.60 ^b^	50.99 ± 21.07 ^a^	50.21 ± 19.6 ^ab^
	RRAW (kg)	7.50 ± 0.98	7.47 ± 0.92	7.55 ± 0.96	7.48 ± 0.98	7.45 ± 0.87	7.62 ± 1.04	7.49 ± 0.98	7.47 ± 0.91	7.57 ± 0.97	7.48 ± 0.98	7.48 ± 0.91	7.57 ± 0.96
	HW^1^ (kg)	23.53 ± 2.39	23.84 ± 2.34	23.15 ± 2.58	23.39 ± 2.33	23.86 ± 2.26	23.27 ± 2.76	23.44 ± 2.36	23.89 ± 2.34	23.18 ± 2.64	23.43 ± 2.37	23.92 ± 2.33	23.16 ± 2.62
	FHW (kg)	5.90 ± 0.62 ^b^	6.04 ± 0.71 ^a^	5.86 ± 0.71 ^b^	5.87 ± 0.62 ^b^	6.06 ± 0.69 ^a^	5.88 ± 0.74 ^b^	5.87 ± 0.62 ^b^	6.07 ± 0.70 ^a^	5.86 ± 0.71 ^b^	5.87 ± 0.62 ^b^	6.07 ± 0.7 ^a^	5.86 ± 0.71 ^b^
	HHW (kg)	3.50 ± 1.09	3.40 ± 0.95	3.38 ± 0.98	3.47 ± 1.08	3.39 ± 0.93	3.44 ± 1.03	3.49 ± 1.09	3.04 ± 0.94	3.39 ± 0.99	3.48 ± 1.09	3.41 ± 0.95	3.40 ± 0.99
	OmW (kg)	4.02 ± 0.76	3.93 ± 0.69	3.88 ± 0.66	3.99 ± 0.75	3.92 ± 0.66	3.94 ± 0.74	4.00 ± 0.75	3.94 ± 0.70	3.90 ± 0.67	4.00 ± 0.75	3.94 ± 0.70	3.90 ± 0.67
	HW^2^ (kg)	1.78 ± 0.31 ^B^	1.88 ± 0.35 ^A^	1.78 ± 0.33 ^B^	1.77 ± 0.31 ^B^	1.89 ± 0.35 ^A^	1.78 ± 0.33 ^B^	1.77 ± 0.30 ^B^	1.88 ± 0.35 ^A^	1.78 ± 0.33 ^B^	1.77 ± 0.31 ^B^	1.89 ± 0.35 ^A^	1.77 ± 0.33 ^B^
	SW (kg)	0.86 ± 0.17	0.87 ± 0.20	0.83 ± 0.20	0.86 ± 0.17	0.87 ± 0.19	0.83 ± 0.20	0.86 ± 0.17 ^b^	0.87 ± 0.20 ^a^	0.82 ± 0.20 ^b^	0.86 ± 0.170 ^b^	0.87 ± 0.20 ^a^	0.82 ± 0.20 ^b^
	LTW (kg)	3.09 ± 0.42 ^B^	3.22 ± 0.49 ^A^	3.14 ± 0.51 ^AB^	3.09 ± 0.42 ^B^	3.24 ± 0.49 ^A^	3.14 ± 0.51 ^AB^	3.09 ± 0.42 ^B^	3.23 ± 0.49 ^A^	3.14 ± 0.52 ^AB^	3.08 ± 0.42 ^B^	3.24 ± 0.49 ^A^	3.14 ± 0.51 ^AB^
	KW (kg)	1.15 ± 0.18 ^B^	1.20 ± 0.22 ^A^	1.16 ± 0.21 ^AB^	1.15 ± 0.18 ^B^	1.20 ± 0.22 ^A^	1.17 ± 0.21 ^AB^	1.15 ± 0.18 ^B^	1.20 ± 0.22 ^A^	1.17 ± 0.21 ^AB^	1.15 ± 0.18 ^B^	1.20 ± 0.22 ^A^	1.17 ± 0.21 ^AB^
	RAW (kg)	4.58 ± 2.70	4.98 ± 2.80	4.81 ± 2.87	4.57 ± 2.67 ^B^	5.11 ± 2.83 ^A^	4.63 ± 2.84 ^AB^	4.53 ± 2.67 ^B^	5.09 ± 2.82 ^A^	4.71 ± 2.84 ^AB^	4.55 ± 2.67 ^B^	5.09 ± 2.83 ^A^	4.67 ± 2.84 ^AB^
	CPW (kg)	0.45 ± 0.09 ^A^	0.44 ± 0.08 ^AB^	0.42 ± 0.08 ^B^	0.45 ± 0.09 ^A^	0.44 ± 0.08 ^AB^	0.42 ± 0.09 ^B^	0.45 ± 0.09 ^A^	0.44 ± 0.08 ^AB^	0.42 ± 0.09 ^B^	0.45 ± 0.09 ^A^	0.44 ± 0.08 ^AB^	0.42 ± 0.09 ^B^
	TaW (kg)	42.22 ± 6.64	42.47 ± 5.98	41.03 ± 5.90	42.12 ± 6.71	42.31 ± 5.70	41.41 ± 6.32	42.27 ± 6.8	42.34 ± 5.74	41.10 ± 6.01	42.16 ± 6.70	42.43 ± 5.87	41.15 ± 5.98
	TeW (kg)	0.63 ± 0.12	0.61 ± 0.13	0.57 ± 0.11	0.67 ± 0.14	0.68 ± 0.15	0.65 ± 0.15	0.67 ± 0.14	0.68 ± 0.15	0.66 ± 0.15	0.67 ± 0.14	0.68 ± 0.15	0.65 ± 0.15
	GFW (kg)	0.93 ± 0.38	0.89 ± 0.34	0.85 ± 0.34	0.91 ± 0.37	0.89 ± 0.34	0.86 ± 0.35	0.92 ± 0.37	0.89 ± 0.34	0.85 ± 0.34	0.92 ± 0.37	0.89 ± 0.34	0.85 ± 0.34
	OxW (kg)	1.34 ± 0.22	1.38 ± 0.26	1.35 ± 0.27	1.33 ± 0.22 ^b^	1.39 ± 0.26 ^a^	1.34 ± 0.26 ^ab^	1.33 ± 0.22 ^b^	1.39 ± 0.27 ^a^	1.34 ± 0.25 ^ab^	1.33 ± 0.22 ^b^	1.40 ± 0.26 ^a^	1.33 ± 0.26 ^b^
	BW (kg)	20.24 ± 3.03	20.23 ± 3.22	19.77 ± 2.98	20.11 ± 3.04	20.27 ± 3.16	19.92 ± 3.12	20.14 ± 3.04	20.28 ± 3.20	19.84 ± 3.00	20.11 ± 3.03	20.32 ± 3.21	19.81 ± 2.99
Fatty acid composition	Myristic acid	0.020 ± 0.017	0.020 ± 0.017	0.025 ± 0.018	0.020 ± 0.017	0.019 ± 0.017	0.027 ± 0.020	0.02 ± 0.017	0.021 ± 0.018	0.025 ± 0.019	0.020 ± 0.017	0.020 ± 0.018	0.025 ± 0.019
	Myristoleic acid	0.002 ± 0.004	0.002 ± 0.004	0.003 ± 0.004	0.002 ± 0.006	0.002 ± 0.004	0.003 ± 0.004	0.002 ± 0.006	0.002 ± 0.004	0.003 ± 0.004	0.002 ± 0.006	0.002 ± 0.004	0.003 ± 0.004
	Palmitic acid	0.265 ± 0.223	0.246 ± 0.179	0.309 ± 0.183	0.264 ± 0.221	0.237 ± 0.174	0.321 ± 0.188	0.261 ± 0.220	0.250 ± 0.180	0.313 ± 0.186	0.264 ± 0.221	0.247 ± 0.181	0.309 ± 0.183
	Palmitoleic acid	0.031 ± 0.044	0.024 ± 0.020	0.031 ± 0.018	0.031 ± 0.044	0.024 ± 0.020	0.031 ± 0.018	0.03 ± 0.043	0.024 ± 0.020	0.031 ± 0.019	0.031 ± 0.044	0.024 ± 0.020	0.031 ± 0.018
	Margaric acid	0.011 ± 0.007	0.011 ± 0.007	0.013 ± 0.008	0.011 ± 0.007	0.011 ± 0.007	0.014 ± 0.008	0.011 ± 0.007	0.012 ± 0.008	0.014 ± 0.008	0.011 ± 0.007	0.012 ± 0.008	0.013 ± 0.008
	Heptadecenoic acid	0.006 ± 0.009	0.004 ± 0.006	0.005 ± 0.005	0.006 ± 0.009	0.004 ± 0.006	0.006 ± 0.005	0.006 ± 0.009	0.004 ± 0.006	0.006 ± 0.005	0.006 ± 0.009	0.004 ± 0.006	0.005 ± 0.005
	Stearic acid	0.183 ± 0.109	0.186 ± 0.119	0.227 ± 0.126	0.182 ± 0.108 ^b^	0.178 ± 0.105 ^b^	0.241 ± 0.140 ^a^	0.181 ± 0.108	0.189 ± 0.120	0.231 ± 0.128	0.182 ± 0.108	0.188 ± 0.120	0.227 ± 0.126
	Oleic acid	0.401 ± 0.541	0.332 ± 0.233	0.388 ± 0.201	0.400 ± 0.535	0.321 ± 0.230	0.401 ± 0.207	0.395 ± 0.530	0.333 ± 0.234	0.393 ± 0.204	0.400 ± 0.535	0.331 ± 0.235	0.387 ± 0.201
	Linoleic acid	0.096 ± 0.025 ^B^	0.101 ± 0.026 ^B^	0.123 ± 0.043 ^A^	0.097 ± 0.025 ^B^	0.098 ± 0.023 ^B^	0.126 ± 0.045 ^A^	0.097 ± 0.024 ^B^	0.101 ± 0.027 ^B^	0.123 ± 0.044 ^A^	0.097 ± 0.025 ^B^	0.100 ± 0.027 ^B^	0.123 ± 0.043 ^A^
	α-linolenic acid	0.004 ± 0.005 ^B^	0.006 ± 0.005 ^AB^	0.009 ± 0.012 ^A^	0.005 ± 0.005 ^B^	0.006 ± 0.005 ^AB^	0.009 ± 0.011 ^A^	0.004 ± 0.005 ^B^	0.006 ± 0.005 ^AB^	0.010 ± 0.012 ^A^	0.005 ± 0.005 ^B^	0.006 ± 0.005 ^AB^	0.009 ± 0.012 ^A^

The different letters (a,b) indicate significant differences among the genotypes (*p* < 0.05); The different letters (A,B) indicate highly significant differences among the genotypes (*p* < 0.01); Mean ± SD, mean ± standard deviation. MBS, marbling score (the score range of marbling is from No. 1 to 9); FCS, fat color score (the score range of fat color is from No. 1 to 7); BFT, backfat thickness; FCR, carcass fat coverage rate; RRAW, rumen, reticulum, and abomasum weight; HW^1^: head weight; FHW, front hoof weight; HHW, hind hoof weight; OmW, omasum weight; HW^2^, heart weight; SW, spleen weight; LTW, lung and trachea weight; KW, kidney weight; RAW, renal adipose weight; CPW, cow penis weight; TaW, tare weight; TeW, testicular weight; GFW, genital fat weight; OxW, oxtail weight; BW, bone weight.

**Table 6:** Association analyses of *SCD1* gene polymorphisms with milk traits in Chinese Holstein cows.

Traits	g.21272246 A>G			g.21272306 T>C			g.21272422 C>T			g.21272529 A>G		
	AA (*n =* 33)	AG (*n =* 36)	GG (*n =* 4)	TT (*n =* 33)	TC (*n =* 36)	CC (*n =* 4)	CC (*n =* 33)	CT (*n =* 36)	TT (*n =* 4)	AA (*n =* 33)	AG (*n =* 36)	GG (*n =* 4)
	Mean ± SD	Mean ± SD	Mean ± SD	Mean ± SD	Mean ± SD	Mean ± SD	Mean ± SD	Mean ± SD	Mean ± SD	Mean ± SD	Mean ± SD	Mean ± SD
Average milk yield (kg/day)	29.38 ± 5.71 ^ab^	26.36 ± 6.44 ^b^	31.86 ± 7.54 ^a^	29.38 ± 5.71 ^ab^	26.36 ± 6.44 ^b^	31.86 ± 7.54 ^a^	29.38 ± 5.71 ^ab^	26.36 ± 6.44 ^b^	31.86 ± 7.54 ^a^	29.38 ± 5.71	26.36 ± 6.44 ^b^	31.86 ± 7.54 ^a^
Milk fat content (%)	4.59 ± 0.38 ^a^	4.53 ± 0.48 ^ab^	4.15 ± 0.47 ^b^	4.59 ± 0.38 ^a^	4.53 ± 0.48 ^ab^	4.15 ± 0.47 ^b^	4.59 ± 0.38 ^a^	4.53 ± 0.48 ^ab^	4.15 ± 0.47 ^b^	4.59 ± 0.38 ^a^	4.53 ± 0.48 ^ab^	4.15 ± 0.47 ^b^
Milk protein content (%)	3.31 ± 0.23	3.29 ± 0.34	3.22 ± 0.25	3.31 ± 0.23	3.29 ± 0.34	3.22 ± 0.25	3.31 ± 0.23	3.29 ± 0.34	3.22 ± 0.25	3.31 ± 0.23	3.29 ± 0.34	3.22 ± 0.25
Milk lactose (%)	4.62 ± 0.26	4.52 ± 0.34	4.42 ± 0.36	4.62 ± 0.26	4.52 ± 0.34	4.42 ± 0.36	4.62 ± 0.26	4.52 ± 0.34	4.42 ± 0.36	4.62 ± 0.26	4.52 ± 0.34	4.42 ± 0.36
Dry matter intake (kg)	13.42 ± 0.92	13.29 ± 1.12	12.94 ± 0.87	13.42 ± 0.92	13.29 ± 1.12	12.94 ± 0.87	13.42 ± 0.92	13.29 ± 1.12	12.94 ± 0.87	13.42 ± 0.92	13.29 ± 1.12	12.94 ± 0.87
Milk urea-nitrogen (mg/dl)	18.7 ± 2.10	18.37 ± 1.74	18.07 ± 3.20	18.7 ± 2.10	18.37 ± 1.74	18.07 ± 3.20	18.7 ± 2.10	18.37 ± 1.74	18.07 ± 3.20	18.7 ± 2.10	18.37 ± 1.74	18.07 ± 3.20

The different letters indicate significant differences among the genotypes (*p* < 0.05); Mean ± SD, mean ± standard deviation.

**Table 7:** Association analysis of SNPs haplotypes combination and carcass traits, meat quality, adipogenesis traits in Chinese Simmental steers.

Traits	Haplotypes Combination				
	H1H1 (*n =* 121)	H1H2 (*n =* 131)	H1H3 (*n =* 5)	H2H2 (*n =* 68)	H2H3 (*n =* 2)
	Mean ± SD	Mean ± SD	Mean ± SD	Mean ± SD	Mean ± SD
LW^1^ (kg)	486.81 ± 58.71	499.98 ± 57.88	477.70 ± 42.14	486.54 ± 64.82	543.00 ± 4.36
CW (kg)	253.68 ± 35.46	262.68 ± 36.55	248.84 ± 30.65	252.54 ± 42.01	294.83 ± 10.80
DP (%)	52.03 ± 2.31	52.43 ± 2.15	51.99 ± 2.70	51.72 ± 2.90	54.29 ± 1.64
BW (kg)	20.21 ± 3.03	20.21 ± 3.18	18.60 ± 2.30	19.84 ± 3.02	20.33 ± 0.58
CL (cm)	140.43 ± 8.13	139.17 ± 8.16	139.60 ± 9.32	137.87 ± 7.95	146.00 ± 5.29
CD (cm)	64.72 ± 3.10	64.21 ± 3.35	63.60 ± 2.88	63.98 ± 3.38	66.67 ± 1.53
CBD (cm)	65.28 ± 3.93	65.37 ± 3.36	64.40 ± 2.79	64.84 ± 3.72	66.50 ± 0.87
HLW (cm)	44.52 ± 2.92	44.82 ± 2.51	43.80 ± 2.80	44.64 ± 2.74	44.17 ± 1.26
HLC (cm)	48.71 ± 4.10	48.94 ± 3.47	48.10 ± 2.30	49.01 ± 3.67	49.67 ± 1.44
HLL (cm)	80.17 ± 4.36	80.05 ± 3.88	80.80 ± 6.41	80.14 ± 3.94	78.17 ± 2.02
WMT (cm)	6.77 ± 0.92	6.96 ± 0.86	6.70 ± 0.72	6.91 ± 0.90	7.67 ± 1.31
TMT (cm)	17.8 ± 1.83	17.88 ± 1.67	17.60 ± 1.47	18.26 ± 1.49	18.37 ± 3.01
HW^1^ (kg)	23.48 ± 2.38	23.82 ± 2.28	22.61 ± 1.97	23.14 ± 2.63	24.49 ± 1.48
FHW (kg)	5.89 ± 0.62	6.05 ± 0.70	5.46 ± 0.50	5.86 ± 0.72	6.21 ± 0.36
HHW (kg)	3.50 ± 1.09	3.37 ± 0.92	3.33 ± 1.08	3.40 ± 0.99	2.94 ± 0.29
TaW (kg)	42.16 ± 6.66	42.18 ± 5.63	43.74 ± 8.10	41.07 ± 6.05	40.94 ± 1.59
BFT (cm)	0.93 ± 0.61	1.10 ± 0.62	1.22 ± 0.99	0.89 ± 0.57	1.60 ± 0.78
FCR%	48.01 ± 21.66 ^b^	51.18 ± 20.67 ^b^	43.20 ± 23.50 ^b^	50.29 ± 19.78 ^b^	73.33 ± 16.07 ^a^
GFW (kg)	0.92 ± 0.37	0.88 ± 0.34	0.78 ± 0.33	0.85 ± 0.35	0.75 ± 0.41
RRAW (kg)	7.49 ± 0.98	7.44 ± 0.86	7.42 ± 1.21	7.58 ± 0.98	7.23 ± 0.89
OmW (kg)	4.01 ± 0.76	3.91 ± 0.67	3.75 ± 0.51	3.88 ± 0.67	3.85 ± 0.52
HW^2^ (kg)	1.77 ± 0.31	1.88 ± 0.35	1.72 ± 0.19	1.77 ± 0.33	2.10 ± 0.02
LW^2^ (kg)	5.87 ± 1.05	6.12 ± 1.10	5.54 ± 0.78	5.84 ± 1.06	6.37 ± 0.59
SW (kg)	0.86 ± 0.18	0.87 ± 0.20	0.80 ± 0.10	0.82 ± 0.20	0.95 ± 0.10
LTW (kg)	3.09 ± 0.42	3.23 ± 0.50	2.93 ± 0.34	3.14 ± 0.51	3.46 ± 0.42
KW (kg)	1.15 ± 0.18	1.21 ± 0.23	1.06 ± 0.19	1.17 ± 0.21	1.08 ± 0.13
RAW (kg)	4.58 ± 2.68 ^b^	5.10 ± 2.80 ^b^	3.28 ± 1.92 ^b^	4.66 ± 2.83 ^b^	8.23 ± 0.89 ^a^
CPW (kg)	0.45 ± 0.09	0.43 ± 0.08	0.46 ± 0.16	0.42 ± 0.09	0.44 ± 0.06
TeW (kg)	0.67 ± 0.14	0.68 ± 0.15	0.69 ± 0.10	0.65 ± 0.15	0.60 ± 0.30
OxW (kg)	1.34 ± 0.23 ^b^	1.39 ± 0.26 ^b^	1.25 ± 0.12 ^b^	1.34 ± 0.26 ^b^	1.75 ± 0.11 ^a^
pH (0 h)	6.33 ± 0.51	6.18 ± 0.50	6.14 ± 0.50	6.17 ± 0.52	5.86 ± 0.19
pH (24 h)	5.60 ± 0.34	5.55 ± 0.31	5.52 ± 0.22	5.49 ± 0.37	5.35 ± 0.23
MBS	5.39 ± 0.72	5.24 ± 0.75	5.60 ± 0.55	5.49 ± 0.68	5.00 ± 1.00
MCS	5.71 ± 1.08 ^b^	5.60 ± 1.09 ^b^	5.20 ± 0.84 ^b^	5.57 ± 1.11 ^b^	7.00 ± 0.00 ^a^
FCS	2.80 ± 1.02	2.75 ± 0.85	2.80 ± 1.10	2.51 ± 1.03	2.33 ± 0.58
REA (cm^2^)	79.36 ± 13.48	81.42 ± 12.79	76.6 ± 5.68	77.71 ± 12.05	74.33 ± 5.77

The different letters indicate significant differences among the genotypes (*p* < 0.05); Mean ± SD, mean ± standard deviation; LW^1^, live weight; CW, carcass weight; DP, dressing percentage; CL, carcass length; CD, carcass depth; HLW, hind legs’ width; CBD, carcass breast depth; HLC, hind legs’ circumference; HLL, hind legs’ length; WMT, waist meat thickness; TMT, thigh meat thickness; HW^1^: head weight; FHW, front hoof weight; HHW, hind hoof weight; TaW, tare weight; BFT, backfat thickness; FCR, carcass fat coverage rate; GFW, genital fat weight; RRAW, rumen, reticulum, and abomasum weight; OmW, omasum weight; HW^2^, heart weight; LW^2^, liver weight; SW, spleen weight; LTW, lung and trachea weight; KW, kidney weight; RAW, renal adipose weight; CPW, cow penis weight; TeW, testicular weight; OxW, oxtail weight; BW, bone weight; pH (0 h), beef pH value after slaughter; pH (24 h), beef pH value 24 h after degassing; MBS, marbling score (the score range of marbling is from No. 1 to 9); MCS, muscle color score (the score range of muscle color is from No. 1 to No. 7); FCS, fat color score (the score range of fat color is from No. 1 to 7); REA, rib eye area.

**Table 8:** Association analysis of SNPs haplotype combination of the *SCD1* gene and fatty acid composition in Chinese Simmental cattle.

Fatty Acid Composition (g/100 g)	Haplotypes			
	H1H1 (*n =* 43)	H1H2 (*n =* 35)	H1H3 (*n =* 3)	H2H2 (*n =* 21)
	Mean ± SD	Mean ± SD	Mean ± SD	Mean ± SD
Myristic acid	0.020 ± 0.018	0.019 ± 0.017	0.017 ± 0.009	0.025 ± 0.019
Myristoleic acid	0.002 ± 0.006	0.002 ± 0.004	0.003 ± 0.004	0.003 ± 0.004
Palmitic acid	0.267 ± 0.226	0.241 ± 0.175	0.199 ± 0.097	0.313 ± 0.186
Palmitoleic acid	0.031 ± 0.045	0.024 ± 0.020	0.023 ± 0.018	0.031 ± 0.019
Margaric acid	0.011 ± 0.007	0.011 ± 0.007	0.009 ± 0.001	0.014 ± 0.008
Heptadecenoic acid	0.006 ± 0.009	0.004 ± 0.006	0.003 ± 0.004	0.006 ± 0.005
Stearic acid	0.184 ± 0.110	0.179 ± 0.106	0.136 ± 0.037	0.231 ± 0.128
Oleic acid	0.405 ± 0.547	0.325 ± 0.233	0.297 ± 0.166	0.393 ± 0.204
Linoleic acid	0.096 ± 0.025 ^B^	0.098 ± 0.023 ^B^	0.099 ± 0.001 ^AB^	0.123 ± 0.044 ^A^
α-linolenic acid	0.004 ± 0.005 ^B^	0.006 ± 0.005 ^AB^	0.005 ± 0.007 ^AB^	0.010 ± 0.012 ^A^
Arachic acid	0.000 ± 0.001	0.001 ± 0.005	0.000 ± 0.000	0.001 ± 0.002
Eicosanic acid	0.001 ± 0.003	0.001 ± 0.002	0.000 ± 0.000	0.001 ± 0.001
Dihomo-γ-linolenic acid	0.010 ± 0.003	0.010 ± 0.003	0.009 ± 0.001	0.010 ± 0.003
Arachidonic acid	0.049 ± 0.013	0.048 ± 0.011	0.058 ± 0.012	0.054 ± 0.017

The different letters indicate significant differences among the genotypes (*p* < 0.05); The different letters (A,B) indicate highly significant differences among the genotypes (*p* < 0.01); Mean ± SD, mean ± standard deviation.

## Data Availability

Data contained within the article.
